# Transforming growth factor-β1 negatively regulates SOCS7 via EGR1 during wound healing

**DOI:** 10.1186/s12964-022-00893-5

**Published:** 2022-06-15

**Authors:** Xiao Feng, Wei Feng, Yu Ji, Tingting Jin, Jingyu Li, Jincai Guo

**Affiliations:** Department of Plastic Surgery, Zhejiang Provincial People’s Hospital, People’s Hospital of Hangzhou Medical College, No. 158 Shangtang Road, Hangzhou, 310014 China

**Keywords:** TGF-β1, SOCS7, EGR1, Keratinocytes, Wound healing

## Abstract

**Background:**

TGF-β1 promotes keratinocyte migration and re-epithelialization of cutaneous wounds during the wound healing process. Decreased SOCS7 expression has been associated with increased healing potential. However, the relationship between TGF-β1 and SOCS7 in wound re-epithelialization remains unclear.

**Objectives:**

To investigate the relationship between TGF-β1 and SOCS7 in the re-epithelialization of keratinocytes during skin wound healing.

**Methods:**

The expression of SOCS7 under different concentrations of TGF-β1 was detected by WB and qPCR. The migration ability of keratinocytes was detected by scratch and Transwell assay. Protein interactions were detected by ChIP and luciferase assay. The effect of SOCS7 on skin healing in mice was detected in animal model.

**Results:**

In this study, we found that SOCS7 was downregulated by TGF-β1 and that overexpression of SOCS7 led to suppression of TGF-β1-induced keratinocyte migration through inhibition of the PI3K/AKT and MEK/ERK pathways. Also, TGF-β1 negatively regulated SOCS7 expression at the transcriptional level through the binding of EGR1 to the EGR1/SP1 overlapping binding sites in the *SOCS7* promoter.

**Conclusion:**

Taken together, our findings show that TGF-β1-induced EGR1 expression is required for repression of SOCS7, which promotes keratinocyte migration and re-epithelialization during wound healing. Finally, our study identifies the TGF-β1/EGR1/SOCS7 pathway as a potential therapeutic target to promote wound healing.

**Video Abstract**

**Supplementary Information:**

The online version contains supplementary material available at 10.1186/s12964-022-00893-5.

## Background

Wound healing is a dynamic and complex process that requires the co-ordination of sequential molecular and cellular events in response to tissue damage [1, 2]. Wound repair begins with haemostasis followed by inflammation, proliferation and remodeling [2]. Re-epithelialization—the process by which a skin wound is resurfaced with new epithelium—is a key event during the proliferation stage of wound healing, and involves the migration and proliferation of keratinocytes [3]. Multiple cytokines and growth factors have been implicated in mediating the re-epithelialization process including transforming growth factor-β1 (TGF-β1) [4–8]. Increased TGF-β1 expression during wound healing [9, 10] has been associated with both inflammation [11] and keratinocyte migration [12]. Interestingly, dysregulation of keratinocyte migration has been linked to a clinical phenotype associated with chronic non-healing wounds [13].

The suppressor of cytokine signaling (SOCS) family has been shown to have a role in wound healing through the regulation of cytokines and growth factors [14, 15]. In mammals, there are eight members of this family [SOCS1 to 7 and cytokine induced SH2-containing proteins (CIS)] [16]. In general, SOCS function mainly through three main domains, namely, the SH2 domain identified as centrally conserved, the N-terminal domain with variable length and divergent sequence, and the carboxy-terminal 40-amino acid module called the SOCS box [17]. Several structurally related members of the eight SOCS family can attenuate cytokine signaling by blocking JAK tyrosine kinase activity, competing with STAT proteins for docking sites and/or binding to their respective target proteins for subsequent proteasome degradation [17]. Previously, overexpression of SOCS3 was found to inhibit keratinocyte migration and proliferation in vitro, and impair wound closure in a transgenic mouse model [18], as well as exacerbate inflammation in the presence of TGF-β1 [19]. A recent study reported increased expression of SOCS3 and SOCS4 in non-healing wounds, while suggesting that decreased SOCS7 expression might be associated with a higher healing prognosis [15].

SOCS7 has been implicated in mediating growth factor responses. For example, SOCS7 was shown to regulate type I insulin-like growth factor (IGF-I) signaling through several mechanisms including interactions with the insulin receptor substrate 1 (IRS-1) and IRS-2 adaptor proteins, which initiate activation of the PI3K/AKT and RAS-REF-MEK/ERK signaling pathways via the IGF-I receptor [20, 21]. In addition, IRS-4 was found to interact with SOCS7, p85, the regulatory unit of PI3K/AKT pathway and PLCγ-1, thereby activating the PKC/ERK pathway [22]. Interestingly, TGF-β1 treatment has been shown to markedly reduce SOCS7 protein expression [23]. However, it remains unclear whether this TGF-β1-mediated decrease in SOCS7 expression contributes to enhanced wound healing potential.

TGF-β1 activates both SMAD and non-SMAD signaling pathways including RhoA-ROCK, MAPK/JNK, MAPK/ERK1/2 and RAS/PI3K to mediate multiple cellular processes including the cell cycle, growth and development, migration and the immune response [24]. TGF-β1 was found to induce cell migration through activation of AKT and ERK1/2 signaling pathways [25, 26], as well as migration of type II endometrial cancer cells through activation of SMAD and ERK1/2 pathways [27], and epithelial-mesenchymal transition and migration of human lung cancer cells through PI3K/AKT and MEK/ERK1/2 signaling [28]. However, the precise mechanisms of action of TGF-β1 in migration and wound healing are not known.

TGF-β1 has been shown to induce expression of transcription factors, including early growth response 1 (EGR1) in human skin fibroblasts [29]. EGR1 has been linked to tissue fibrosis and wound healing in multiple studies [29–32]. EGR1 and the transcription factor, specificity protein 1 (SP1) have been shown to compete for binding on their overlapping binding sites on the *EGR1* promoter [33], as well as the *PPARγ* [34] and *NGX6* [35] promoters. Binding of EGR1 to its own promoter was found to downregulate EGR1 expression via MAPK pathways [36]. Furthermore, EGR1 has been shown to be a transcriptional regulator of SOCS1 [37]. The relationship between EGR1 and SOCS7 remains unclear, as does the role of EGR1 in mediating TGF-β1-dependent keratinocyte migration.

Here, we found that TGF-β1 downregulated SOCS7 expression, while SOCS7 overexpression suppressed TGF-β1-induced keratinocyte migration through inhibition of the PI3K/AKT and MEK/ERK pathways. TGF-β1 down-regulated SOCS7 at the transcriptional level by promoting the binding of EGR1 to the EGR1/SP1 overlapping binding site in the *SOCS7* promoter. Thus, our findings demonstrate that EGR1 promotes TGF-β1-induced keratinocyte migration in vitro and re-epithelialization during wound healing in vivo through repression of SOCS7.

## Methods

### Cell culture

HaCaT cells were obtained from the Type Culture Collection of Chinese Academy of Sciences (Shanghai, China). Cells were cultured in DMEM (Gibco, Waltham, MA, USA) containing 10% fetal bovine serum (FBS; Gibco) and 1% penicillin/streptomycin (Gibco) at 37 °C in a humidified atmosphere of 5% CO_2_.

### Reverse transcription‑quantitative polymerase chain reaction (RT‑qPCR)

Total RNA was extracted using TRIzol (Invitrogen, Waltham, MA, USA). RNA was reverse-transcribed into cDNA using RevertAid First Strand cDNA Synthesis Kit (Thermo Fisher Scientific, Waltham, MA, USA) according to the manufacturer’s instructions. Relative mRNA levels were normalized to *Gapdh* and calculated using the 2^−ΔΔCT^ method. The following primer sequences were used: SOCS7 Fp: 5′-CCTCAGTTTCCGATCACAGGGTA-3′, Rp: 5′-TGGACAGGAGTTGGTGGCAGT-3′; EGR1 Fp: 5′-AAAGTTTGCCAGGAGCGAT-3′, Rp: 5′-GGGGGAACAGAGGAGTACG-3′; and GADPH Fp: 5′-TCAAGAAGGTGGTGAAGCAGG-3′, Rp: 5′-TCAAAGGTGGAGGAGTGGGT-3′.

### Transfection of keratinocytes

HaCaT cells were cultured to 80% confluency, then transfected with overexpression plasmids or siRNAs using Lipofectamine 2000 (Invitrogen) for 24 h. For the SOCS7 overexpression experiments, the SOCS7 coding sequence (CDS) was cloned and inserted into the pcDNA3.1 plasmid (Invitrogen). For the knockdown experiments, the following siRNAs were obtained from GeneChem (Shanghai, China): SOCS7 siRNA target sequence, 5′-CCAGTGTCCCGATTCAGCAATGTCA-3′; EGR1 siRNA target sequence, 5′-GCGATGAACGCAAGAGGCATACCAA-3′; and SP1 siRNA target sequence, 5′-GAGAGGCCATTTATGTGTACCTGGT-3′. Control siRNA (siR-ctrl) was purchased from GeneChem.

### Western blot analysis

Total protein was extracted from cells/tissue using RIPA lysis buffer (Beyotime Biotechnology, Shanghai, China) containing protease inhibitor cocktail (Roche, Basel, Switzerland). The protein concentration was determined using a BCA protein assay kit (Beyotime) according to the manufacturer’s instructions. Protein (30 μg) was separated by 10% SDS‑PAGE, then transferred to a polyvinylidene fluoride membrane. The membrane was washed twice with 5% fat-free milk in PBST (PBS containing 0.05% Tween-20) for 1 h at room temperature, then incubated at 4 °C overnight with primary antibodies against EGR1 (1:500, Santa Cruz Biotechnology, Dallas, TX, USA), TGF-β1 (1:1000, Santa Cruz Biotechnology), SOCS7 (1:1000, Santa Cruz Biotechnology), p-AKT (1:1000, Abcam, Cambridge, UK), AKT (1:1000, Abcam), ERK1/2 (1:1000, Abcam), p-ERK1/2 (1:1000, Abcam) and GAPDH (1:2500, Abcam). Membranes were washed twice the following day, then incubated with HRP-conjugated secondary antibodies for 1 h at room temperature. Protein bands were visualized with a chemiluminescence detection system (Pierce, Rockford, IL, USA). Bands were normalized to GAPDH.

### Scratch assay

HaCaT cells were cultured to 90% confluency. A sterile 200 μl pipette tip was used to create a scratch, then cells were cultured in DMEM for a further 24 h. The scratch gap was visualized using a phase contrast microscope. Wound healing rates were calculated by ImageJ software.

### Transwell assay

Cell migration was assessed using the Transwell migration assay. Briefly, HaCaT cells were cultured in serum-free medium for 24 h. Cells were placed in the upper chamber of the Transwell plate in serum-free medium, while medium containing 10% FBS was placed in the lower chamber. Cells were incubated for 24 h at 37 °C. Migrated cells were fixed in methanol, stained with 0.1% crystal violet and visualized using a light microscope (ECLIPSE Ts2, Nikon).

### Luciferase reporter assay

The ~ 600 bp upstream from the transcription start site of the human *SOCS7* gene harboring the promoter region was amplified by PCR using the following primers: forward, 5′-CAACAGACAGCTCACCGCC-3′ and reverse, 5′-GCAGTTCCGAGGGTCCCG**-**3′, then cloned into the pGL4-Basic vector (Promega, Madison, WI, USA). To construct mutant plasmids, the putative S/E binding sites (386–373: CCCCCGCCCCCCTC; 257–244: CTCCCTCCCTCTCC; 129–116: CCACCGCCCCCGGG; 108–95: CCCCCGCCGCCACC) were deleted (pGL4-ΔE/S).

HaCaT cells were cultured in 12-well plates for 24 h, then treated with TGF-β1 (10 ng/ml) for 24 h. Luciferase activity was measured using a Dual Luciferase Reporter Assay Kit (Promega). For knockdown experiments, the luciferase reporter construct was co-transfected with EGR1 siRNA or SP1 siRNA for 48 h.

### Chromatin immunoprecipitation (ChIP)

The ChIP assay was carried out as follows. Briefly, treated cells were cross-linked using 1% formaldehyde at room temperature for 10 min. Cross-linked chromatin was sonicated to reduce the DNA fragments to 200–1000 bp. Soluble chromatin was centrifuged for 10 min at 14 000 rpm at 4 °C, and 1% of the supernatant was saved as input DNA. Chromatin samples were pre-cleared using protein G agarose, then immunoprecipitated at 4 °C overnight with primary antibodies against SP1 (Cell Signaling Technologies, Danvers, MA, USA) or EGR1 (Santa Cruz Biotechnology). Immunocomplexes were recovered using the ssDNA/protein G agarose slurry, washed with TE buffer, then incubated in elution buffer for 15 min at 25 °C, followed by heating at 65 °C for 6 h. DNA fragments were obtained by phenol/chloroform extraction, then precipitated with ethanol. Immunoprecipitated DNA samples were analyzed by PCR using the following primer pair: forward, 5′- AGCCGCCTCCTTGGCTATG-3′ and reverse, 5′- GACCCACGTTGCGGAACAC-3′. PCR products were separated by electrophoresis and a 151 bp product was detected.

### Wound healing animal model

C57BL/6 J mice (6 weeks old) were obtained from Shanghai SLAC Laboratory Animal Co., Ltd (Shanghai, China). All animal experiments were approved by the Institutional Review Board of Zhejiang Provincial People's Hospital (Hangzhou, China). Full thickness wounds (1 × 1.5 cm^2^) were created on the dorsal skin and left open. Mice were treated with control siRNA (siR-ctrl), siR-EGR1, siR-SOCS7 or siR-EGR1 + siR-SOCS7 delivered topically by pipette into the wound cavity (1 μg/wound) every second day for 12 days. The wound surface area was measured at various time points (days 0, 3, 7, 10 and 14) following wounding, and wound closure was calculated relative to the surface area of the initial wound. ImageJ software (National Institute of Health) was used to measure the wound surface area.

Mice were killed on day 14 using CO_2_ asphyxiation. The wounds together with 2 mm surrounding skin were dissected and fixed in 4% paraformaldehyde. Sections (4 μm thick) were cut and stained with hematoxylin and eosin (H&E) to determine the degree of re-epithelialization.

### Immunohistochemistry

Tissue samples taken from the wound area and 2 mm surrounding skin on days 0 and 7 after wound healing were fixed in formalin and embedded in paraffin. Frozen Sects. (4 μm thick) were cut, fixed in 4% paraformaldehyde for 15 min, then incubated with Triton X-100 for 10 min at room temperature. Sections were incubated with primary antibodies against EGR1 (1:100, Santa Cruz Biotechnology), TGF-β1 (1:150, Santa Cruz Biotechnology) or SOCS7 (1:100, Santa Cruz Biotechnology) at 4 °C overnight. The following day, samples were incubated with HRP-conjugated second antibodies for 30 min at room temperature, followed by the DAB Detection System kit (Servicebio, Wuhan, China). Cell nuclei were counterstained with hematoxylin. Samples were visualized using light microscopy.

### Statistical analysis

The two-tailed Student’s *t* test was used to compare two different groups. One way ANOVA followed by Bonferroni post-hoc analyses (where appropriate) was used to compare differences between more than two groups. Data are expressed as mean ± standard deviation (SD) of three independent experiments, each performed in triplicate. A p value < 0.05 was considered to be statistically significant.

## Results

### SOCS7 is downregulated by TGF-β1 and mediates TGF-β1-induced keratinocyte migration and wound healing

**Fig. 1:**
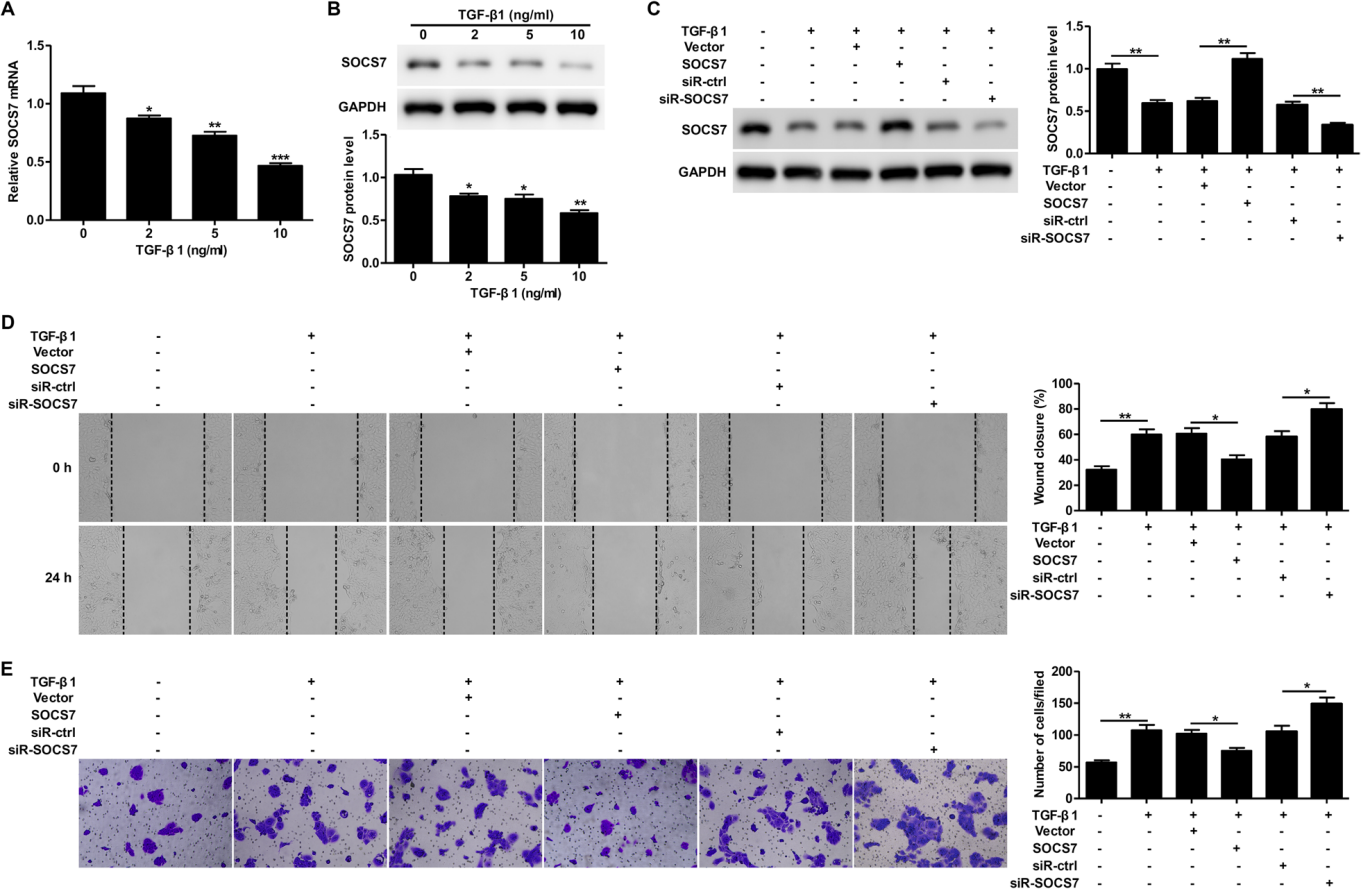
SOCS7 is downregulated by TGF-β1 and mediates TGF-β1-induced keratinocyte migration and wound healing. **A, B**. SOCS7 mRNA and protein levels in TGF-β1-treated HaCaT cells were analyzed by RT-PCR (**A**) and western blotting (**B**), respectively. **C–E**. HaCaT cells were transfected with Vector or SOCS7 plasmids, and scrambled siRNA (siR-ctrl) or SOCS7 siRNA (siR-SOCS7) for 24 h, then treated with TGF-β1 (10 ng/ml) for 24 h. SOCS7 protein levels were analyzed by western blotting (**C**). The scratch‐wound assay was performed to analyze the effect of SOCS7 on TGF‐β1‐dependent cell migration. Representative images (left) and quantification of data showing the percentage of wound closure (right) (**D**). The Transwell migration assay was carried out to determine the effect of SOCS7 on TGF‐β1-dependent cell migration. Representative images showing crystal violet staining (left) and quantification of data showing the number of migrated cells/field (right) (**E**). Data are given as mean ± standard deviation (SD) of three independent experiments. *P < 0.05; **P < 0.01; ***P < 0.001

TGF-β1 treatment was found to down-regulate SOCS7 mRNA (Fig. 1A) and protein (Fig. 1B) expression levels in HaCaT cells in a dose-dependent manner. Overexpression or silencing of SOCS7 using a SOCS7 overexpression plasmid or siR-SOCS7 in TGF-β1-treated HaCaT cells was found to increase or decrease SOCS7 protein expression, respectively (Fig. 1C). Since downregulation of SOCS7 has been implicated in wound healing [15], we next examined the effects of SOCS7 overexpression and knockdown on TGF-β1-dependent cell migration using a scratch assay. We found that TGF-β1 significantly increased keratinocyte migration in vitro (Fig. 1D), while overexpression of SOCS7 decreased TGF-β1-induced migration and SOCS7 knockdown led to a more pronounced effect (Fig. 1D). Similarly, we found using a Transwell migration assay that SOCS7 overexpression inhibited TGF-β1-induced migration of HaCaT cells, while siR-SOCS7 treatment promoted further migration (Fig. 1E). Taken together, these data show that TGF-β1 downregulates SOCS7 expression, and that downregulation of SOCS7 promotes cell migration.

### SOCS7 regulates TGF-β1-induced keratinocyte motility through the PI3K/AKT and MEK/ERK pathways

**Fig. 2:**
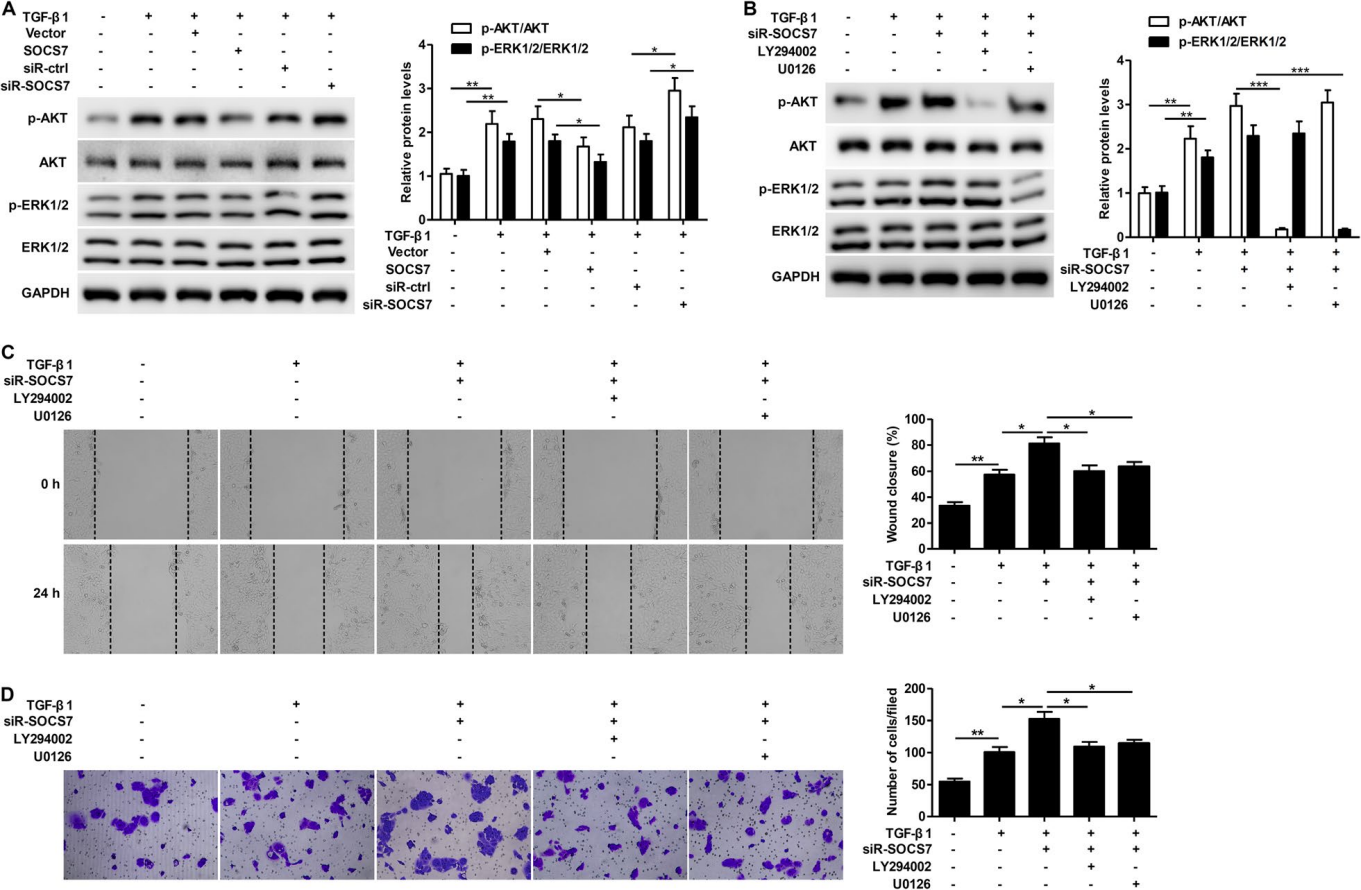
SOCS7 regulates TGF-β1-induced keratinocyte motility through the PI3K/AKT and MEK/ERK pathways. **A**. HaCaT cells were transfected with Vector or SOCS7 plasmids, and scrambled siRNA (siR-ctrl) or SOCS7 siRNA (siR-SOCS7) for 24 h, then treated with TGF-β1 (10 ng/ml) for 24 h. Protein expression levels of phosphorylated ERK1/2 (p‑ERK1/2), total ERK, phosphorylated AKT (p‑AKT) and total AKT were analyzed by western blotting (**A**). **B–D**. HaCaT cells were transfected with SOCS7 siRNA (siR-SOCS7) for 24 h, followed by LY294002 (10 μM) or U0126 (10 μM) for 1 h, then finally TGF-β1 (10 ng/ml) treatment for 24 h. Protein expression levels of phosphorylated ERK1/2 (p‑ERK1/2), total ERK, phosphorylated AKT (p‑AKT) and total AKT were analyzed by western blotting (**B**). Wound healing was assessed using the scratch‐wound assay. Representative images (left) and quantification of data showing the percentage of wound closure (right) (**C**). Cell migration was assessed using the Transwell migration assay. Representative images showing crystal violet staining (left) and quantification of data showing the number of migrated cells/field (right) (**D**). Data are given as mean ± standard deviation (SD) of three independent experiments. *P < 0.05; **P < 0.01; ***P < 0.001

To determine the mechanism of action of SOCS7 in mediating TGF-β1-induced keratinocyte motility, we examined the protein expression levels of p-AKT, AKT, p-ERK and ERK in TGF-β1-treated HaCaT cells in the presence of a SOCS7 overexpression plasmid or siR-SOCS7. Treatment with TGF-β1 significantly increased both p-AKT/AKT and p-ERK/ERK expression levels compared to untreated cells (Fig. 2A). Overexpression of SOCS7 in TGF-β1-treated cells led to a significant decrease in p-AKT/AKT and p-ERK/ERK expression, while SOCS7 knockdown resulted in significantly increased p-AKT/AKT and p-ERK/ERK levels (Fig. 2A). We next silenced SOCS7 expression in TGF-β1-treated HaCaT cells, which had been treated with the PI3K inhibitor, LY294002 or MEK inhibitor, U0126. As expected, LY294002 significantly blocked p-AKT/AKT, while U0126 significantly blocked p-ERK/ERK (Fig. 2B). Treatment with either LY294002 or U0126 reversed the siR-SOCS7-induced increase in wound healing (Fig. 2C) and migration (Fig. 2D) in TGF-β1-treated cells, suggesting that SOCS7 regulates TGF-β1-induced keratinocyte motility through the PI3K/AKT and MEK/ERK pathways.

### TGF-β1 regulates SOCS7 expression at the transcriptional level via EGR1

**Fig. 3:**
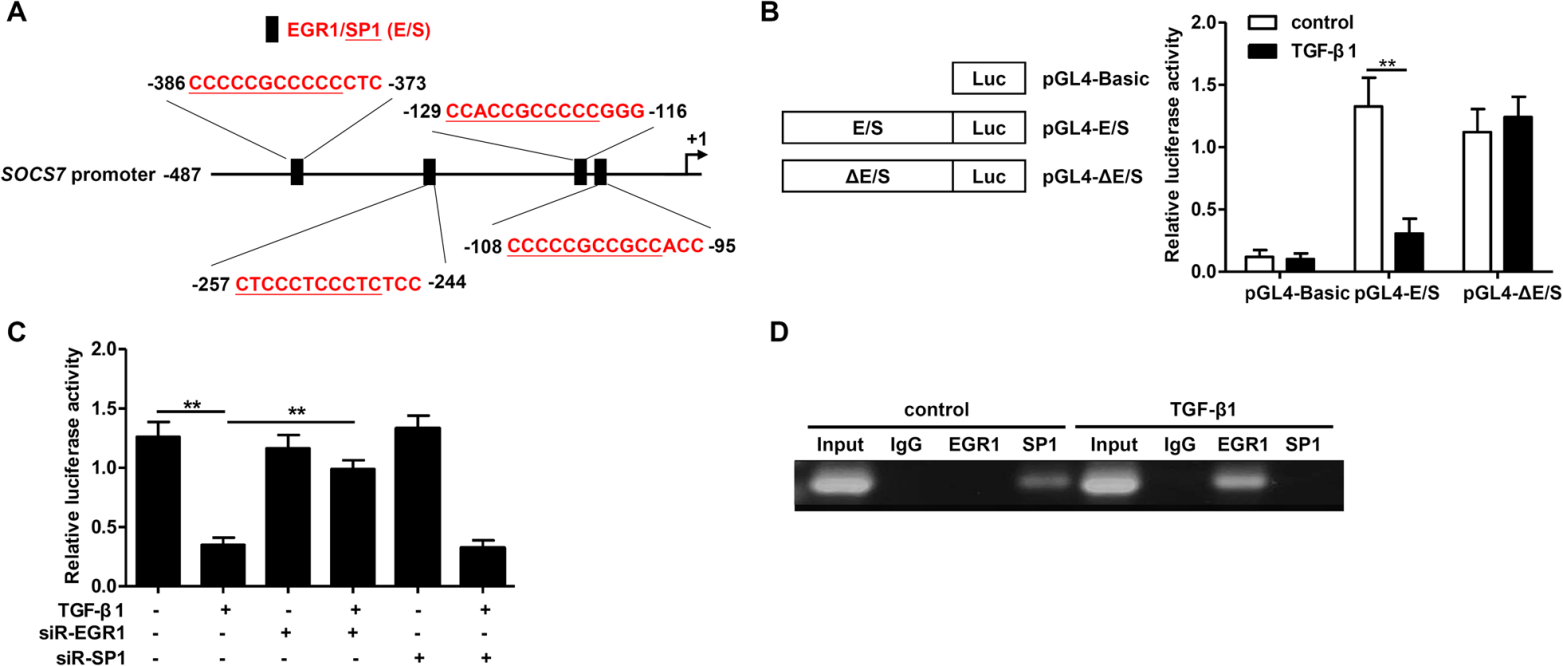
TGF-β1 regulates SOCS7 expression at the transcriptional level via EGR1. **A**. Schematic diagram showing the locations of the predicted overlapping binding sites for EGR1 (in red) and SP1 (in red and underline) (E/S sites) on the *SOCS7* promoter. E/S sites were predicted using JASPAR and Animal Transcription Factor databases. **B**. HaCaT cells were transfected with luciferase constructs under control of a *SOCS7* promoter encompassing either the wild-type sequence (pGL4-E/S) or a mutant with the putative binding S/E sites deleted (pGL4-ΔE/S), or with pGL4 and a renilla control plasmid for 24 h. Then, cells were treated with TGF-β1 (10 ng/ml) for 24 h and the luciferase activity was measured. **C**. HaCaT cells were transfected with EGR1 siRNA (siR-EGR1) or SP1 siRNA (siR-SP1) for 24 h, stimulated with or without TGF-β1 (10 ng/ml) for 24 h, then the luciferase activity was measured. **D**. HaCaT cells were treated with or without TGF-β1 (10 ng/ml) for 24 h. Representative ChIP assay of TGF-β1-mediated EGR1 and SP1 binding to the *SOCS7* promoter is shown. Data are given as mean ± standard deviation (SD) of three independent experiments. *P < 0.05; **P < 0.01; ***P < 0.001

Using the JASPAR and Animal Transcription Factor databases, we predicted the locations of the overlapping binding sites for the transcription factors EGR1 and SP1 on the *SOCS7* promoter region (Fig. 3A). To determine whether these transcription factors regulated SOCS7 mRNA expression, we used a luciferase assay with luciferase constructs under the control of a *SOCS7* promoter with either the wild type (pGL4-E/S) or mutant (pGL4-ΔE/S) putative binding EGR1/SP1 (E/S) sites. Luciferase activity in TGF-β1-treated HaCaT cells was significantly decreased in wild-type but not mutated constructs (Fig. 3B), indicating that TGF-β1 mediates transcriptional regulation of SOCS7 via these EGR1/SP1 binding sites. To determine whether EGR1 or SP1 was required for TGF-β1-induced repression of SOCS7, we knocked down EGR1 (siR-EGR1) or SP1 (siR-SP1) in TGF-β1-treated cells. We found that knockdown of EGR1 blocked the TGF-β1-mediated reduction in luciferase activity, while siR-SP1 had no effect (Fig. 3C). In addition, using a ChIP assay, we confirmed that TGF-β1 promoted binding of EGR1, but not SP1 to the *SOCS7* promoter (Fig. 3D). Thus, taken together our findings indicate that TGF-β1-dependent repression of SOCS7 is mediated by EGR1.

### Knockdown of EGR1 prevents TGF-β1-induced keratinocyte motility

**Fig. 4:**
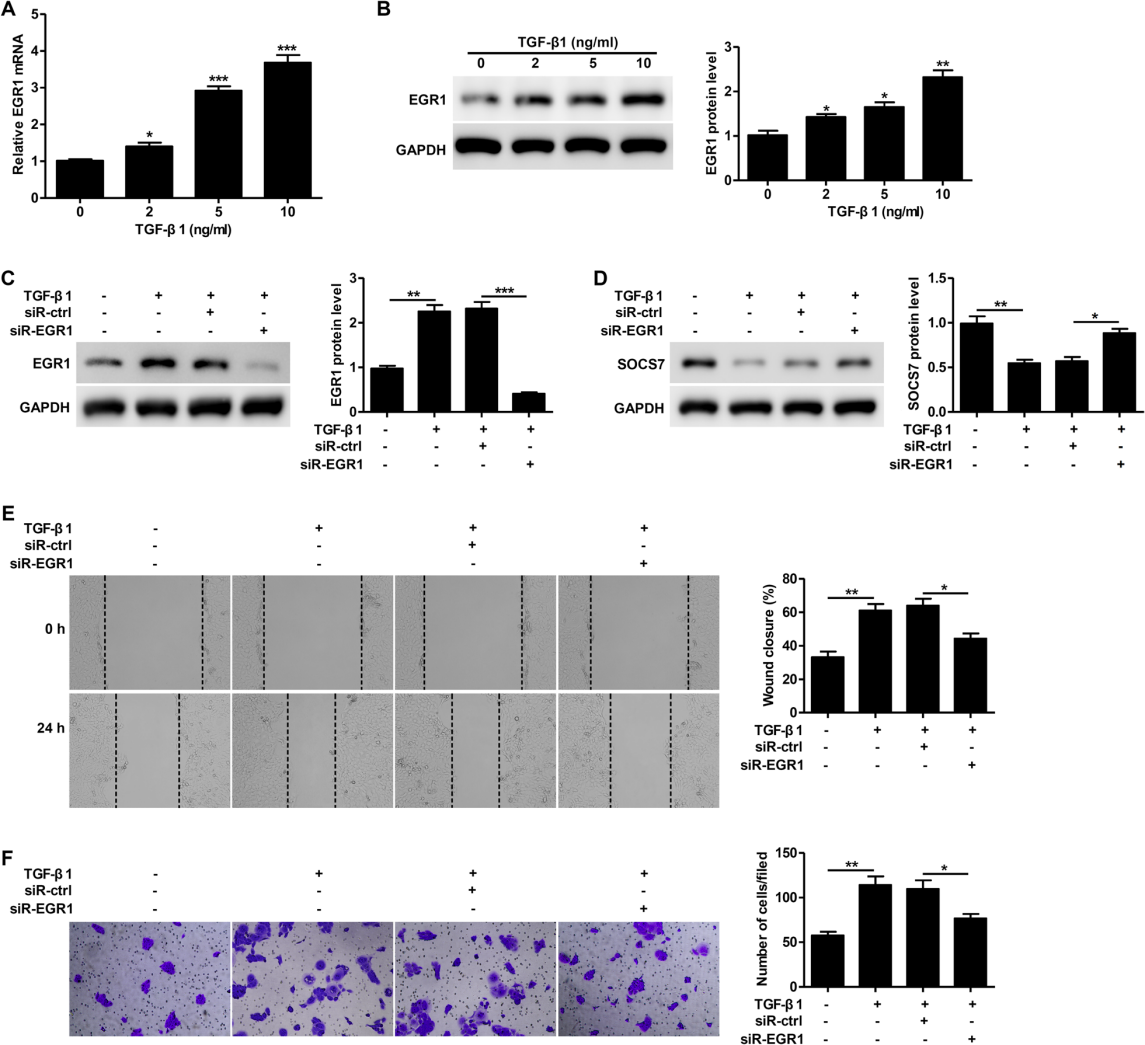
Knockdown of EGR1 prevents TGF-β1-induced keratinocyte motility. **A–B**. EGR1 mRNA and protein levels in TGF-β1-treated HaCaT cells were analyzed by RT-PCR (**A**) and western blotting (**B**), respectively. **C–F**. HaCaT cells were transfected with scrambled siRNA (siR-ctrl) or EGR1 siRNA (siR-EGR1) for 24 h, then treated with TGF-β1 (10 ng/ml) for 24 h. EGR1 protein levels were analyzed by western blotting (**C**). SOCS7 protein levels were analyzed by western blotting (**D**). The scratch‐wound assay was used to examine the effect of EGR1 knockdown on TGF‐β1‐dependent wound healing. Representative images (left) and quantification of data showing the percentage of wound closure (right) (**E**). Cell migration was assessed using the Transwell migration assay. Representative images showing crystal violet staining (left) and quantification of data showing the number of migrated cells/field (right) (**F**). Data are given as mean ± standard deviation (SD) of three independent experiments. *P < 0.05; **P < 0.01; ***P < 0.001

We found that treatment with TGF-β1 increased EGR1 mRNA (Fig. 4A) and protein (Fig. 4B) expression in a dose-dependent manner, while siR-EGR1 treatment significantly blocked this TGF-β1-induced increase in EGR1 (Fig. 4C). The TGF-β1-mediated reduction in SOCS7 expression was rescued by knockdown of EGR1 (Fig. 4D). The TGF-β1-mediated increase in keratinocyte migration as measured by both the scratch (Fig. 4E) and Transwell (Fig. 4F) assays was significantly reduced in the presence of siR-EGR1. Taken together, these findings suggest that TGF-β1 induces keratinocyte motility through EGR1.

### TGF-β1-induced keratinocyte motility is mediated through EGR1 and is partially dependent on the repression of SOCS7

**Fig. 5:**
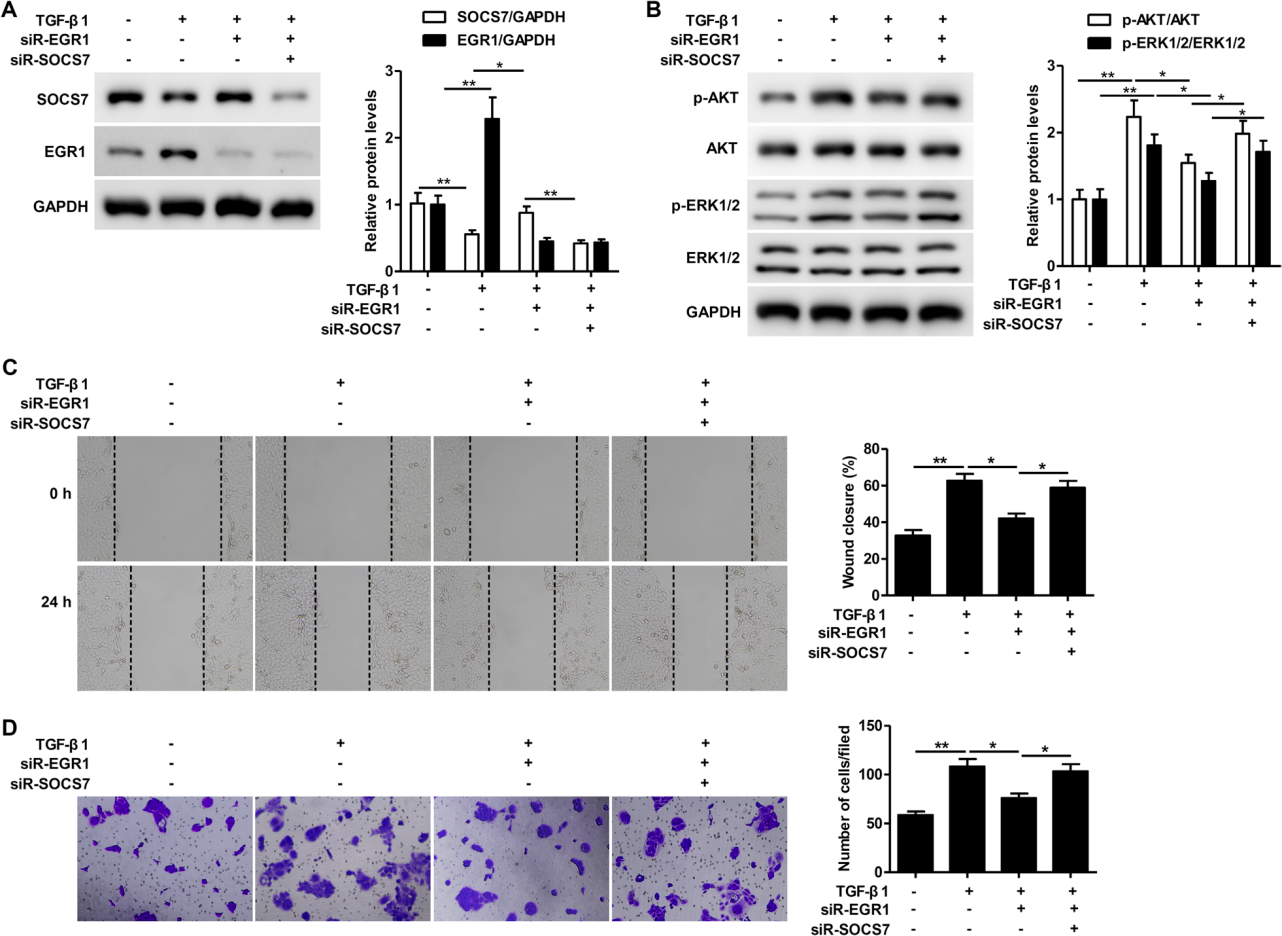
TGF-β1-induced keratinocyte motility is mediated through EGR1 and is partially dependent on the repression of SOCS7. **A–D**. HaCaT cells were co-transfected with EGR1 siRNA (siR-EGR1) or SOCS7 siRNA (siR-SOCS7) for 24 h, then treated with TGF-β1 (10 ng/ml) for 24 h. **A**. SOCS7 and EGR1 protein levels were analyzed by western blotting. **B**. Protein expression levels of phosphorylated ERK1/2 (p‑ERK1/2), total ERK, phosphorylated AKT (p‑AKT) and total AKT were analyzed by western blotting. **C**. Wound healing was assessed using the scratch‐wound assay. Representative images (left) and quantification of data showing the percentage of wound closure (right). **D**. Cell migration was assessed using the Transwell migration assay. Representative images showing crystal violet staining (left) and quantification of data showing the number of migrated cells/field (right). Data are given as mean ± standard deviation (SD) of three independent experiments. *P < 0.05; **P < 0.01; ***P < 0.001

We next sought to determine whether EGR1 promoted TGF-β1-induced keratinocyte motility through SOCS7. Knockdown of EGR1 inhibited the TGF-β1-dependent repression of SOCS7 protein expression (Fig. 5A). Silencing EGR1 expression reduced the TGF-β1-induced activation of the PI3K/AKT and MEK/ERK pathways, while silencing both EGR1 and SOCS7 expression led to partial restoration of p-AKT/AKT and p-ERK/ERK levels (Fig. 5B). Similarly, knockdown of EGR1 reduced TGF-β1-induced wound healing and migration, while knockdown of both EGR1 and SOCS7 restored keratinocyte motility (Fig. 5C, D). Taken together, these findings demonstrate that EGR1 mediates its effect on TGF-β1-induced wound healing and migration through partial repression of SOCS7.

### EGR1 promotes re-epithelialization during wound healing in vivo via partial repression of SOCS7

**Fig. 6:**
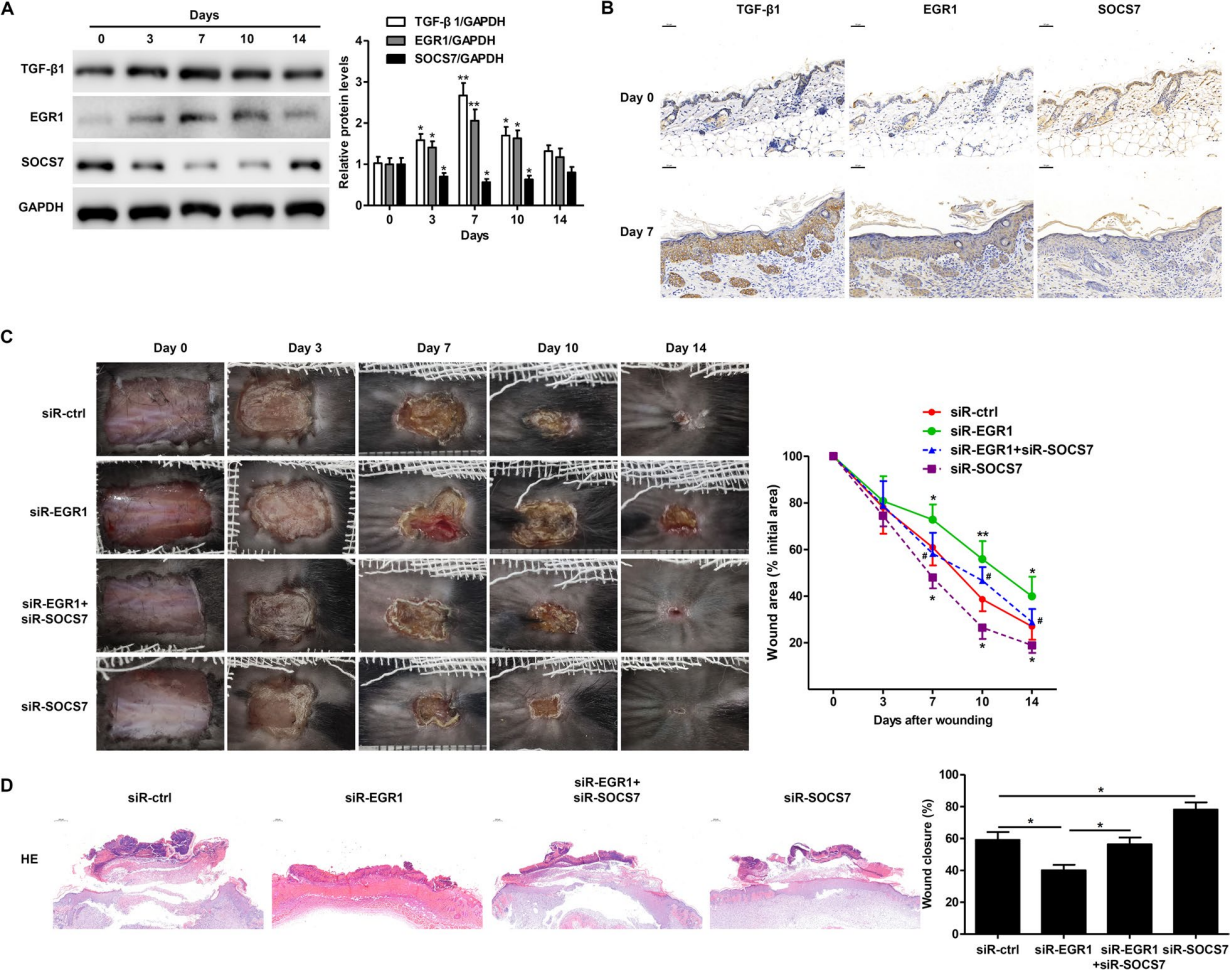
EGR1 promotes re-epithelialization during wound healing through partial suppression of SOCS7 expression. **A**. Western blot analysis of TGF-β1, EGR1, and SOCS7 protein expression levels in C57BL/6 J mouse skin tissue at the wound edge at the indicated days after wounding. **B**. Representative immunohistochemical images of TGF-β1, EGR1, and SOCS7 staining in C57BL/6 J mouse skin tissue at the wound edge at days 0 and 7 post-wound-injury. **C**. Representative images showing wound healing over the course of 14 days post-wounding in mice treated with scrambled controls (siR-ctrl), EGR1 siRNA (siR-EGR1), or SOCS7 siRNA (siR-SOCS7) (left). Quantification of wound healing was carried out by calculating the wound area as a percentage of the initial wound area (right). **D**. Representative images of H&E-stained sections from C57BL/6 J mouse skin tissue on day 7 post-wound-injury showing the degree of re-epithelialization (left panel). The percentage of re-epithelialization (% wound closure) was determined using ImageJ software (right panel). Data are given as mean ± standard deviation (SD) from five mice. *P < 0.05; **P < 0.01; ***P < 0.001

We next used a mouse model of wound healing to determine whether EGR1 and SOCS7 had a role in re-epithelialization in vivo. We found that TGF-β1 and EGR1 protein levels were significantly higher in skin tissue at the wound edge during the first ten days of wound healing with maximum levels observed at day 7 (Fig. 6A). In contrast, SOCS7 protein expression was significantly decreased during the first ten days with lowest expression levels observed at day 7 (Fig. 6A). These findings were confirmed by immunohistochemical analysis (Fig. 6B). We next examined the effects of EGR1 and/or SOCS7 knockdown on wound healing in vivo. We found that SOCS7 knockdown promoted wound healing, while EGR1 knockdown led to slower wound healing (Fig. 6C). Co-treatment with siR-EGR1 partially inhibited the effects of siR-SOCS7, confirming the relationship between EGR1 and SOCS7. H&E staining also revealed significantly increased re-epithelialization in siR-SOCS7-treated mice and significantly reduced wound closure in siR-EGR1-treated mice (Fig. 6D). Our findings suggest that EGR1 promotes TGF-β1-induced keratinocyte migration and re-epithelialization during wound healing through the partial repression of SOCS7.

## Discussion

Here, we found that TGF-β1 down-regulated SOCS7 at the transcriptional level by promoting the binding of EGR1 to the overlapping binding site for EGR1 and SP1 in the *SOCS7* promoter. TGF-β1-dependent SOCS7 repression promoted keratinocyte migration and was associated with decreased PI3K/AKT and MEK/ERK signaling. Thus, our findings demonstrate that TGF-β1-induced EGR1 expression is required for repression of SOCS7 during wound healing in vivo.

The SOCS family of proteins have been implicated in wound healing through the regulation of cytokine and growth factor signaling [14, 15]. Although downregulation of SOCS1-6 has been reported to be involved in the wound healing process, the role of SOCS7 remains unclear [15]. Here, we show that TGF-β1 significantly downregulated SOCS7 in a dose-dependent manner. Furthermore, we demonstrate that SOCS7 knockdown had a dramatic effect on the wound healing process in vivo. Thus, our data suggest that SOCS7 has a critical role in wound healing and could be a potential therapeutic target during tissue repair.

Multiple studies have described a role for TGF-β1 in wound healing [5–7, 9, 11]. Although multiple signaling pathways have been implicated in mediating this TGF-β1-dependent wound healing [24], the precise mechanism remains unclear. Here, we found that the PI3K/AKT and MEK/ERK signaling pathways were involved in TGF-β1-dependent wound healing. Previous studies have implicated both the PI3K/AKT [38–40] and MAPK/ERK1 signaling pathways in wound healing [41–44]. Here, silencing of EGR1 decreased TGF-β1-induced activation of the PI3K/AKT and MEK/ERK pathways, suggesting that TGF-β1 mediated its effect on these signaling pathways via EGR1. However, knockdown of both SOCS7 and EGR1 led to partial restoration of the TGF-β1-induced PI3K/AKT and MEK/ERK activation, suggesting that SOCS7 was downstream of these signaling pathways. Interestingly, overexpression of SOCS2 in skin keratinocytes has been shown to promote cell migration and increase cutaneous wound healing through the EGF/MEK/ERK pathway [44].

Mechanistically, we found that downregulation of SOCS7 by TGF-β1 was mediated by the transcription factor EGR1. Previous studies have shown that TGF-β1 induces expression of EGR1 [29], and that EGR1 is associated with wound healing [29–32]. We identified several EGR1/SP1 binding sites in the *SOCS7* promoter and showed that they were required for TGF-β1-induced downregulation of SOCS7. Further, we demonstrated that EGR1 but not SP1 was required for repression of SOCS7, consistent with other genes including PPARγ [34] and NGX6 [35]. We also found that knockdown of EGR1 prevented TGF-β1-induced keratinocyte migration, and that this effect was mediated through the PI3K/AKT and MEK/ERK pathways. Finally, we showed that silencing of both EGR1 and SOCS7 led to partial restoration of p-AKT/AKT and p-ERK/ERK levels, suggesting that the TGF-β1/EGR1/SOCS7 axis is a central regulatory pathway in wound healing.

Our data suggested that SOCS7 would be an attractive therapeutic target to promote wound healing in vivo. Indeed, we found that knockdown of SOCS7 led to enhanced wound healing in a mouse model and that silencing EGR1 partially reversed this effect. Thus, the EGR1/SOCS7 pathway is an important regulator of wound healing in vivo and is a viable target for the development of therapies to enhance tissue repair. Inhibiting the function of SOCS proteins, either by blocking known functional domains with small molecules or inhibiting known SOCS protein activators [45] may provide a novel method to promote re-epithelialization and wound healing.

## Conclusion

In summary, our findings identify EGR1 and SOCS7 as novel regulators of the TGF-β1-mediated wound healing process, and indicate that the TGF-β1/EGR1/SOCS7 pathway could be a potential therapeutic target to promote wound healing.

## Data Availability

The dataset supporting the conclusions of this article are included within the article.

## Abbreviations

TGF-β1: Transforming growth factor-β1
SOCS: Suppressor of cytokine signaling
IGF-I: Type I insulin-like growth factor
IRS-1: Insulin receptor substrate 1
EGR1: Early growth response 1
SP1: Specificity protein 1
CDS: Coding sequence
ChIP: Chromatin immunoprecipitation
H&E: Hematoxylin and eosin
